# The transition of care from farm-based daycare for people with dementia: The perspective of next of kin

**DOI:** 10.1080/17482631.2023.2228047

**Published:** 2023-06-23

**Authors:** Liv Bjerknes Taranrød, Øyvind Kirkevold, Ingeborg Pedersen, Siren Eriksen

**Affiliations:** a Norwegian National Centre for Ageing and Health, Vestfold Hospital Trust, Tønsberg, Norway; b Faculty of Medicine, University of Oslo, Oslo, Norway; c Centre for Age Related Functional Decline and Diseases (AFS) at Innlandet Hospital Trust, Hamar, Norway; d Department of Health Sciences in Gjøvik, Norwegian University of Science and Technology (NTNU), Gjøvik, Norway; e Department of Public Health Science, Ås, Norway, Norwegian University of Life Sciences, Ås, Norway; f Department of Bachelor of Nursing, Lovisenberg Diaconal University College, Oslo, Norway

**Keywords:** Next of kin, family caregiver, dementia, farm-based daycare, transition of care in the municipality, support, qualitative, interviews

## Abstract

**Purpose:**

The aim of the present study was to explore the next of kin’s experiences with the transition for people with dementia from a farm-based daycare (FDC) to another service in the municipality.

**Methods:**

The study has a qualitative, descriptive design. Eight semi-structured interviews with next of kin were conducted. The data were analysed in accordance with content analysis.

**Results:**

Through the analysis three main categories were developed: (1) Bearing the burden, (2) Being in transition, and (3) Feeling supported. The transition period was highly stressful for next of kin due to the exacerbation of their relatives’ dementia symptoms. The next of kin focussed on optimizing the everyday lives of their relatives with dementia, even at the expense of their own well-being. Most participants experienced support from FDC, healthcare services and their informal network.

**Conclusions:**

The study contributes important insights into the next of kin’s experiences. Good quality service, close dialog, information, and support between the different part in the transition process, can be useful for the further development of services with good quality and to reduce the negative effects of care on next of kin.

## Introduction

Informal care is considered the cornerstone of dementia care. Most people with dementia live in their own homes and have at least one next of kin, often a spouse or another family member, caring for them (Association, 2022). Norwegian authorities have adopted a policy whereby people with dementia should continue to live at home, if possible, with support from the municipal healthcare system and their next of kin (Norwegian Ministry of Health and Care Services, 2018, 2020). The municipalities are obliged to provide health and social services to their citizens in accordance with care needs corresponding to the lowest effective level of care (Norwegian Directorate of Health, 2015). According to regulations, they should offer different measures to those next of kin with particularly burdensome care tasks; these measures may include training and guidance, respite and care benefits (Norwegian Ministry of Health and Care Services, 2011). It is the municipalities that determine how they organize these services and which services they offer each next of kin; thus, the services offered will vary (Norwegian Directorate of Health, 2019b). In Norway, nearly 90% of the municipalities have coordinators or multidisciplinary teams with competence in dementia who provide useful support and information about this condition and the services available to next of kin and those with dementia (Norwegian Directorate of Health, 2019a).

Dementia affects a person’s cognitive abilities and activities of daily living as the disease progresses. Therefore, people with dementia will need increasingly comprehensive care and intervention measures (Livingston et al., 2020). In addition, many will experience neuropsychiatric symptoms during the course of their disease, and this aspect of dementia is the most important predictor of burden for next of kin, leading also to transitions to higher levels of care (Toot et al., 2017; Wergeland et al., 2015). Caring for a person with dementia often lead to impaired physical and mental health, sleep disturbances, reduced social well-being and an increasing burden on caregivers as well as a financial strain (Adelman et al., 2014; Del-Pino-Casado et al., 2018). A lack of social support for both people with dementia and their next of kin, a low-quality dyadic relationship, and increased caregiver burden or health challenges, among other factors, accelerate a shift in care service (Maffioletti et al., 2019; Toot et al., 2017; Verbeek et al., 2015).

Next of kin living with a spouse/partner with dementia are particularly vulnerable to negative consequences of caregiving compared to other next of kin (Johansson et al., 2021; L. Taranrød et al., 2020). Although caring for people with dementia is often associated with negative experiences, many next of kin also report experiencing positive aspects of caregiving such as a sense of personal accomplishment, gratification, feelings of mutuality, an increase of family cohesion, and a sense of personal growth and purpose (Yu et al., 2018). Moreover, experiences of negative aspects are not necessarily obstacles to positive experiences; both are possible (Johansson et al., 2022; Yu et al., 2018). Positive caregiving experiences are more probably to arise when the next of kin experiences both personal and social affirmation in the caregiving giving role (Yu et al., 2018).

Daycare service (DC) facilitated for people with dementia is considered an important service for home-dwelling people with dementia (Norwegian Ministry of Health and Care Services, 2020). In Norway, most DCs services are located in a healthcare institution or service centre for older adults and is often referred to as regular DC (Ibsen et al., 2018; Norwegian Directorate of Health, 2019a). Farm-based daycare service (FDC) is one type that represents a daycare service complementary to regular DC (Ibsen et al., 2018). The majority of Norwegian FDCs have people with dementia in early stage as their main target group. In FDC there are more men compared to DC, the participants are younger and more often live with a spouse or partner (Ibsen et al., 2019; Rokstad et al., 2017). In Norway, FDC and DC have similarities with regard to the organization, daily structure, and the number of health education personnel (Ibsen et al., 2018). Compared to DC, FDC have fewer participants per day; more numbers of employees per participants, have open fewer days per week and differ in type of care environment (Ibsen et al., 2018). FDC actively uses the agricultural environments to facilitate for a range of activities for the participants with dementia, connected to the farm buildings, gardens, animals, and outdoor areas (Ibsen et al., 2018). Being physical active, spending time outdoors and being in social interactions with other people are the core components of the service offered at FDC (Ibsen et al., 2018).

For those being next of kin to a relative attending FDC, respite is closely connected to the well-being of their relative with dementia and the quality and content of the service. The FDC is described as person centred care of good quality. The staff at FDC was an important support for the next of kin along the trajectory of the progression of dementia and they felt included (L. B. Taranrød et al., 2021).

Both FDC and DC aim to facilitate meaningful activities in a safe environment and to improve the quality of life for people with dementia while providing respite for their next of kin. The intent of these services is to support people with dementia, as well as their next of kin, so that those with dementia can continue to live in their own home as long as possible (Norwegian Ministry of Health and Care Services, 2020).

In this study, a “transition” is understood as a shift between FDC to another service in the municipality. Next of kin typically experience transitions several times because the person with dementia will require more comprehensive care during the dementia course (Cranwell et al., 2018). In Norway, a person with dementia who stops attending an FDC most often transitions to nursing-home care. Some enter a DC located in a healthcare institution, whereas a few stops attending any form of daycare service. Transition of care may indicate a person’s need for more-comprehensive care than FDC can provide or a need for other and not so challenging surroundings (Ibsen et al., 2020). Any transition that involves a change in the physical environment can lead to increased physical and mental strain for both the person with dementia and his or her next of kin (Afram et al., 2015; Caldwell et al., 2014; Eika et al., 2014). Prior to a transition to nursing home, the caregiving that the next of kin provides often increases significantly but not the services from the municipalities (Vossius et al., 2015). Several studies have shown that spouses may face a considerably challenging in their caregiver role and might experience guilt, grief, and multiple losses, including loss of a partner, loss of identity as a couple, and loss of personal freedom during such a transition (Afram et al., 2014; Chan et al., 2013; Jacobson et al., 2015).

There are few studies of next of kin to people with dementia attending a FDC and to our knowledge, no studies investigating the next of kin’s experience of the transition from FDC to higher level of care. Thus, the aim of our study was to explore the next of kin’s experience of the transition process for people with dementia from FDC to another municipality service.

## Method

### Design

This study has a qualitative, descriptive design and we used interviews as method to gain a deeper understanding of the nature and meaning of the experiences of the next of kin (Kvale & Brinkmann, 2015). We have made efforts to be transparent and have analysed the data according to the well-recognized method of content analysis (Graneheim & Lundman, 2004; Lindgren et al., 2020).

The present study is part of a larger project, Farm-Based Day Care Services for People with Dementia: Quality Development through Interdisciplinary Collaboration, a prospective study with a multi-method approach (Eriksen et al., 2019). The participants were unknown for the authors prior to the project.

### Participants and recruitment

We invited nine people from the larger project to participate in the present study. They were next of kin to persons with dementia who had recently stopped attending FDC and transferred to another service in the municipality. One potential participant withdrew from the study before the interviews were conducted; thus, the sample comprised eight participants. The inclusion criterion was being the next of kin to a person with dementia who had recently stopped attending an FDC and transitioned to another service in the municipality. The participants were recruited from six different FDCs in various regions of Norway through the leader (farm provider) of FDC for what amounted to a purposeful sample reflecting both sexes and different ages (Polit & Beck, 2021). The participants included two men and six women ranging in age from 51 to 77 years and they were next of kin to six men and two women with dementia, at the age of 71 to 83 years old. All the eight participants had a long-term relationship with their relative with dementia. Seven of them were spouses living with the person with dementia, and one was an adult child cohabiting a parent with dementia. Four participants were working, and four were retired. The relatives with dementia attended FDC one to 5 days a week before they stopped attending FDC.

### Data collection

The individual interviews were conducted between June 2017 and January 2018 and took place from 6 weeks to 7 months after the relative with dementia had left FDC. The participants selected the site of the interview; six were interviewed in their homes, one was interviewed via video conference and one by phone. The interviews were based on dialogue and steered by an interview guide with open-ended questions (Kvale & Brinkmann, 2015). In the interviews, the interviewer addressed topics related to the care situation and the next of kin’s experiences prior to their relative’s discontinuing FDC attendance and up to the time they obtained another service in the municipality, examples of questions are shown in the interview guide (Table 1). All the interviews were conducted by the first author (LBT). The interviews lasted from 30 to 90 min and were recorded; then, LBT and a research assistant transcribed the interviews verbatim.

**Table 1.** Examples of the questions in the interview guide.

Please describe the care situation before your relative discontinued attending the FDC.
How did you experience the transition period?
How did you and your relative with dementia participate in the decision to discontinue FDC and apply for a new service in the municipality?
How did you experience the support from the FDC, and the municipality’s healthcare service?
How did you experience the support from close family members and friends?

### Data analysis

The transcribed interviews were subjected to a manifest level of content analysis following the guidelines of Graneheim and Lundman (2004) and led by LTB in collaboration with the co-authors. To support the coding and organization of the data, NVivo 12 Pro was used (QSR International Pty Ltd, 2020). We have strived to describe the analysis process in detail to facilitate transparency in the study (Graneheim & Lundman, 2004; Lindgren et al., 2020). Each interview was identified as a unit of analysis, and the material was evaluated with a focus on manifest levels of content during a five-step analysis (Graneheim & Lundman, 2004).

In step one, the interview transcripts were read several times to obtain an overview of the material. In step two, the text was divided into meaning units, also described as “words, sentences or paragraphs containing aspects related to each other through their content and context” (Graneheim & Lundman, 2004, p. 106), and condensed units were created. In step three, the meaning units were extracted and labelled with codes. In step four, codes were compared based on differences and similarities and grouped into six subcategories. For the fifth and last step, the six subcategories were clustered and grouped into three main categories. Differences in interpretations of the data were discussed by the authors until consensus was reached. An example of the process appears in Table 2.

**Table 2.** Example of the analyse process.

Meaning unit	Condensed meaning unit	Codes	Sub-category	Category
*I felt that I hit the wall, I was so tired. Lack of sleep and I had to trail after him virtually everywhere, so I was simply unable to go to work.*	The next of kin was unable to go on, lack of sleep and having to trail after the person with dementia made her unable to work.	Exhaustion Lack of sleep Sickness leave	Experience physical and mental strain	**Bearing the burden**
*Yes, sometimes I get rather frustrated. I shouldn’t be angry with her for her inability to do something. I know that the disease is the reason why she cannot do it. When I tell her like for the sixth time what to do, I get frustrated – I may even snap at her – and she reacts, then I feel sort of bad.*	The next of kin occasionally feels frustrated about the situation, but isn’t allowed to be angry, since the disease is the reason why persons with dementia fail to cope with things. The next of kin feels bad.	Feelings of frustration, sadness, guilty conscience		
*It was about coping with practical matters in general, getting dressed, grooming, he didn’t know how to wash himself (…) I couldn’t go out, I needed someone to be there when leaving the house, if only for shopping.*	The person with dementia has problems with practical matters, getting dressed, grooming. Could not leave the person with dementia alone in the house.	Take over tasks Constricted	Committed to the situation	
*I was focused on us making it work, and it did (…) I do have some obligations in various places, and I gave them all up. I just resigned from the world.*	The next of kin was focused on finding a solution and succeeded but had to renounce all other obligations.	Renounced all other obligations		

### Ethical aspects

Before the interview, each participant received oral and written information about the study and provided written consent. The interviewees were assured that their participation was voluntary, that they could withdraw from the study at any time and that their personal confidential information was guaranteed. The participants were informed that the researcher was a registered nurse (RN). The study was reported to the Norwegian Centre for Research Data (No. 49799) and conducted in accordance with the Declaration of Helsinki (World Medical Association Declaration of Helsinki, 2004). The anonymized written transcripts of the audio recorded interviews are stored in a secured research server at Norwegian National Center for Aging and Health. The audio-recorded interviews and coding list linked to the participants are deleted as required by the Norwegian Centre for Research Data (No. 49799). The participants did not receive any financial or other benefits from participating in the study.

## Results

All participants expressed that they were satisfied with the quality of FDC, with how their relatives with dementia had been cared for, and with their experience of support. There were great variations in how long the persons with dementia had attended FDC, from 6 to 45 months. Two of them had been transferred to a DC facility in a nursing home immediately after leaving FDC, and two had moved into a nursing home. The four other persons with dementia moved back and forth several times between different facilities before being offered permanent residence in a nursing home.

During the transition period when the functioning of the people with dementia decreased and their need for care increased, the beneficial aspects of respite declined especially for the spouses. The transition from FDC to another municipal service is a process developing and taking place in a context and the participants described their situation in light of this context.

The analysis resulted in three main categories: (1) Bearing the burden; (2) Being in transition; and (3) Feeling supported (Table 3).

**Table 3.** Main categories and subcategories.

Categories	Bearing the burden	Being in transition	Feeling supported
Subcategories	Experiencing physical and mental strain	Making decisions	Support from healthcare service
	Committed to the situation	Preparing for admission to new service	Support from family and friends

### Bearing the burden

The experiences of “Bearing the burden” had two important perspectives: *Experiencing physical and mental strain* and *Committed to the situation*. The situation leading to the transition lasted from a few months up to a year before the relative with dementia was offered another service. Although, for some the transition went unplanned and quickly due to hospitalization of the relative with dementia.

### Experiencing physical and mental strain

All the participants described behavioural changes of the relatives with dementia several months before the transition. Examples of these included episodes of wandering, anxiety, agitation, aggression, and/or passivity. Some participants also experienced the exacerbation of somatic diseases and a decline in ADL for their relative with dementia. All these changes caused both physical and mental strain, especially for the spouses, such as feelings of physical exhaustion, often caused by lack of sleep, and very limited opportunities to leave their homes:

*His disease worsened around Christmas last year, and he slept very poorly at night, so we slept something like 2 to 3 hours a night. At the same time, he started to become unpredictable in his behaviour. So, he couldn’t be left alone unsupervised.* (Spouse)

The participants described feelings of guilt, frustration over becoming impatient and sadness at witnessing how dementia had changed their relative with dementia. Several spouses reported being afraid at times due to unpredictable episodes of agitation and threats. If the agitation occurred at the FDC, the relative with dementia was returned home. Episodes with agitation led to less respite than the spouses might otherwise have: “(…) I felt that I hit the wall. I was so tired. Lack of sleep. I had to follow him virtually everywhere, so I was simply unable to go to work” (Spouse). For some of the working spouses, the care situation occasionally caused absence from work. Balancing jobs with caring for relatives also posed challenges for their daily schedules. Several participants managed to mobilized members of their social network or planned with a home-nursing service or FDC to be able to sort out their daily schedule.

### Committed to the situation

All the participants were concerned with the well-being of their relatives with dementia. They described a feeling of commitment to caring for their relative. The spouses especially stated that even if their own well-being was compromised, they felt a great responsibility. Their only option was to endure the situation. One spouse said, “I just had to live in the situation”, and another stated, “I was focussed on us making it work, and it did. (…) I do have some obligations in various places, and I gave them all up. I just resigned from the world.” The spouses also described their relatives increasingly need for help, they worried about how long they would be able to provide care alone.

### Being in transition

All the participants were aware that their relative’s attendance at the FDC was limited. The process of being in transition was described from two perspectives: *Making decisions* and *Preparing for admission to a new service.*

### Making decisions

*Making decisions* referred to decisions about service options, first, to discontinue the FDC and, second, to apply for other services. In the process of ending FDC, most participants felt that there had been a good dialogue about the care situation with the FDC staff. However, the final decisions were made by the municipality healthcare service, and the participants felt that they and their relatives with dementia had little influence regarding this decision. One spouse described an absence of dialogue:

*We didn’t consider leaving the FDC at all; it was the multidisciplinary team, or the service office, that decided that she couldn’t continue. We didn’t ask for a new service; we just wanted to continue, and no contact was made. (…) We received a letter—that’s all.* (Spouse)

Regardless of when the application for nursing home placement had been made, all the participants described the decision as agonizing but utterly necessary, given the need for care of their relatives with dementia and their own capacity to provide further care at home. At the same time, they expressed sadness that their relative had to leave FDC. “One can say, it was very sad that she could not continue because she thrives there” (Spouse).

### Preparing for admission to a new service

The perspective of *preparing for admission to a new service* refers to the period when the decision to discontinue the FDC was made and before a new care option had been decided. During the transition process some of the participants and their relatives with dementia were invited to visit the new place. Being introduced to the new DC or nursing home and given an opportunity to become familiar with the service was experienced as positive. One spouse said, “It was a month in advance; we were up there (DC) talking to the manager, and we felt welcome”. The participants with relatives who were transitioned directly from FDC to a nursing home where the relatives had stayed earlier for a short time were also content since they were familiar with the place and the staff.

Several of the participants reported that their relative with dementia experienced an exacerbation of his or her somatic illness or neuropsychiatric symptoms, which resulted in a short stay in hospital before transitioning to a nursing home. In some cases, the relative with dementia stayed at several wards at different nursing homes before he or she was granted a permanent stay. This was experienced as extraordinarily demanding, exhausting, and mostly beyond their control. They also experienced a lack of information flow between the various units, and they had to provide the same information about the person with dementia over and over again. Other participants experienced that their relatives with dementia had been placed on a waiting list and had to accept the first vacancy: “There was no choice. You had to take the nursing home service that was available. The municipality decided” (Spouse). One participant reported that the relative was left without any kind of service for several weeks. Others did not receive any information about care options even if they had asked for it.

### Feeling supported

The feeling of support was important to the participants and took mainly place from two sources: *Support from healthcare services* and *Support from family and friends*.

### Support from healthcare services

The participants experienced being met with understanding of their situation and offered some practical help from the healthcare service.

Most participants described that, before the transition of their relative with dementia, they had experienced a valuable dialogue with the head of the FDC and the multidisciplinary team. A participant stated, “Over the last two months, she (the head of the FDC) has been very positive, very supportive, and made all the necessary provisions” (Adult child). The participants also emphasized that their relative with dementia had enjoyed the time at the FDC. The participants therefore felt sad when their relative had to leave this service. Only a few participants were offered extra respite days during the transition period. Several highlighted that the multidisciplinary team had been an important source of support regarding information, guidance and has helped them to cope with the care situation; additionally, some had received help from the home-nursing service.

All participants stated that, it was pivotal that their relatives with dementia received high-quality care after leaving FDC and they had actively sought information about suitable care options for their relatives. When information was insufficient, further frustration occurred. Despite some negative experiences, most of the participants reported that the support from the healthcare system in the municipality, had been important for them in their caregiving situation.

### Support from family and friends

Some of spouses experienced losing relationship with friends because of the situation of their relative with dementia, but most participants stated that they had relied on certain family members or friends for support and expressed gratitude for the care and support that they had received. The participants described having received practical support—for instance, accompanying the person with dementia on doctor’s visits or staying with him or her when the participants needed to complete necessary chores or errands outside home. One spouse described how she had mobilized her family and friends to help with practical tasks regarding the daily schedule so that she could continue her employment:

*(…) the problem is that I start work at half-past eight and the daycare does not open so early; we tried to make it work. One day a week, he was at FDC; three days a week, he was at a daycare in a nursing home; and on the fifth day, my older relative took care of him or our friends that he was confident with (…). All the shifting was too much for him and for me. It did not work; then, we were offered a permanent place before Easter* (Spouse)

Several participants reported that they felt emotionally supported by family and friends and that they could share their worries and feelings about the care situation: “I have a very good friend, a colleague of mine, who called me two or three times a week. You find out who your good friends are—and which ones aren’t” (Spouse). On the matter of being open and sharing thoughts about their situation, the participants differed. Several of the spouses expressed that they had not given their families details about their husband/wife`s situation or their own experience of the caregiving situation. They felt much alone. Others had chosen an opposite solution and shared information with their children. This was seen as an advantage in their difficult situations.

## Discussion

In this study, we found that the transition process was characterized by a longer period of physical and mental strain due to behavioural changes of the relatives with dementia, but the participants were committed to bearing the burden. In relation to the transition process several fundamental and necessary, but agonizing decisions had to be made. In addition, they had to prepare themselves and their relatives with dementia for admission to a new service. Support from health care personnel, family and friends was experienced as especially important.

The transition process was experienced as an exhausting period for the participants. The participants, especially the spouses, described feelings of burden, loss, and grief, and a feeling of uncertainty in relation to their living situation. The negative consequences that caregiving had on their work and social lives were not unexpected and aligned with literature about being a caregiver during the course of dementia (Eika et al., 2014; Evans & Lee, 2014; Association, 2022).

All the participants in the present study, focused on the well-being of the relatives with dementia often on the cost of their own well-being. The care situation gave the participants, especially the spouses, few possibilities to re-energize and recover from the caregiving. Despite the lack of enough respite, our findings provide knowledge about the participants’ views of the service offered. They highlighted the care quality and the support they received from FDC staff as important for them and their relatives with dementia, but the support was not enough to prevent the heavy burden of care that most of the next of kin experienced in the transition process. Even though, the Norwegian authorities have focused on supporting the next of kin in their caregiver roll there is still a lack of available services to meet the next of kin’s needs for respite and support (Granbo et al., 2019; Norwegian Ministry of Health and Care Services, 2020).

Even though the main target group for FDC most often is people in an early stage of dementia, two-third of those who stopped attending FDC moved into a nursing home (Ibsen et al., 2018, 2020). In our study we saw that the relatives with dementia stayed in FDC even though their dementia condition was in a sever stage. The fact that FDC have fewer participants per day and a staff that has the possibility to provide a variety of activities. This could improve the possibility for offering individually tailored services (Ibsen et al., 2020). I In addition, the next of kin experienced a good dialog with the staff and this could also be a factor for why the persons with dementia stayed at FDC for so long.

Our study found that the multidisciplinary team had provided significant support, offering information and guidance in the trajectory of dementia. Yet, in the process of transition, some reported that the healthcare service failed to give them appropriate information about different services, and this was a bit unexpected. One might wonder if the healthcare service personnel are lacking an adequate overview of the services offered by the municipality, despite the fact that the municipality is obliged to provide information and services according to citizens’ needs (Norwegian Directorate of Health, 2015). The participants in our study and their relatives with dementia did not participate in the final decision to leave FDC, the municipality healthcare service made that decision. Despite this, the participants described being engaged in the dialogue about the decision and felt supported by the staff in FDC. Somewhat surprisingly, one of the dyads reported having received only a letter stating that the relative with dementia could no longer attend the FDC, without any dialogue with the healthcare service. This finding indicate that the healthcare services have to improve their routines for supporting next of kin and fulfil their obligations for supporting the caregivers (Norwegian Ministry of Health and Care Services, 1999).

Our findings indicate that, in a planned transition where the next of kin and the person with dementia were given an opportunity to become familiar with the new service and staff before the actual transition, were a positive experience. This is also shown by Tolo Heggestad and Førde (2021) who described the importance of a planned transition and of becoming acquainted with a service as critical factors for a good transition and a positive experience for both the person with dementia and their next of kin. In contrast, when the transition was unplanned and involved several relocations for the person with dementia before he or she made a permanent move, the process and this period were experienced as highly stressful and often included a lack of information, continuity, or predictability in the service to come. The Norwegian guidelines for dementia care (2017) state that deviations like a transition process need to be addressed in order to improve the system and its services. In the transition process, the municipal healthcare service considers the next of kin’s voice as crucial (Tolo Heggestad & Førde, 2021), and our findings suggest that the municipality health care service must be strengthened in order to support the next of kin and their relatives with dementia throughout the course of dementia and particularly when the dementia progresses dementia and the burden of care increases.

Our participants emphasized the importance of support from both the family and healthcare services. Even if, for a variety of reasons, our participants did not involve the family in all of the difficult care situations and decisions regarding the relative with dementia, they valued their support. This might indicate that the participants, in particular the spouses, wished to protect both their family members and the person with dementia from being involved in difficult situations. Alternatively, they may have viewed the situation as a marital or child/parent matter that did not require the involvement of family. Another study on the next of kin of people with dementia attending FDC found that perceived support positively affected both the burden of care and the quality of life (L. Taranrød et al., 2020). In general, social support is considered beneficial not only for maintaining mental health and psychological well-being but also for reducing the risk of depression (Norwegian Directorate of Health, 2021).

## Methodological considerations

In qualitative studies *trustworthiness* describes the quality of the study. To ensure trustworthiness of our study, we have attempted to describe the process openly (Lincoln & Guba, 1985). Lincoln and Guba (1985) emphasize five essential elements of trustworthiness: *credibility, dependability, confirmability, transferability, and authenticity*.

One author (LTB), who is a registered nurse with clinical experiences and a research interest in the care situation for the next of kin of people with dementia, conducted alle the interviews. Polite and Beck (2021) claim that pre-understanding may prevent us from seeing new and previously unknown aspects of our research. To ensure *confirmability* and *credibility* of the study, the interviewer had to be aware of her pre-understanding. When preparing for the study LBT was therefore interviewed by the last author (SE) about her experiences and pre-understanding of the field. The interview was recorded, and LBT listened to it several times to reflect on her pre-understanding and form a conscious relationship with her prior assumptions and perspectives. Throughout the interviews, LTB sought to be as objective as possible and carefully listen to the interviewees’ stories without letting her pre-understanding interfere.

Our participants described their experiences with the transition process of their relatives with dementia from FDC to another municipal service in depth and in their own words. Although member checking has not been carried out by having the participants read the transcribed interviews afterwards, we have attempted to stay true to the participant’s description of the process to ensure *dependability*. The analysis process was conducted in an open dialogue among the authors, discussing meanings, similarities, and differences in understanding the data. The three co-authors are researchers with many years of experience in various aspects of dementia care and dementia care research. Each step of the analysis is described in detail, and the presentation of results in our article is supported by quotes to be transparent and describe the participants various experiences.

The sample is small, with data from eight participants, but the participants represent different FDCs, and regions of Norway and they differ in age, gender, and relationship with their relative with dementia. Polit and Beck (2021) state that including participants with various experiences increases the possibility of shedding light on the research question from a variety of aspects and, thereby, increases its *credibility* and the *authenticity*. Among the eight participants, seven were spouses and one was an adult child of a person with dementia, all added important knowledge to the study. This said, it could possibly have strengthened the study’s credibility to have several adult children or participants with other relations to a person with dementia. For our study it was not possible to recruit such participants. Another limitation was that the interviews were conducted retrospectively and up to 7 months after the relative with dementia had left FDC. We intended to recruit participants shortly after the transition, but we experienced that it was too early and that the next of kin needed some time to process the experience before they were able to talk about it with a researcher.

We believe that the findings elucidate important experiences of next of kin to a relative with dementia that could be *transferable* to others in a similar situation in a different context.

## Conclusion

To highlight the experiences of next of kin during the transition process, politicians and healthcare services must understand, acknowledge, and emphasize the next of kins and the persons with dementia situation and needs. Our findings highlight the importance of good quality service, close dialog, information, and support between the different part in the transition process and can be useful for the further development of services with good quality and to reduce the negative effects of care on next of kin. A planned transition and an opportunity to become acquainted with the new service before the transition seemed to be key factors in experiencing a smooth transition. To ensure continuity and ease the burden of care throughout the course of dementia and in the transition process, the next of kin and the person with dementia should have a contact person who knows them and their situation. This person should act as a coordinator and facilitate the transition process until a permanent stay has been established. Our findings suggest that there is a need for good routines in communication between services in the transition process and with the next of kin. We suggest that the dialog about transitions start early in the process that ensure predictability for people with dementia, the next of kin and the healthcare system.
